# Development and externally validated prediction model of individualization of FSH starting dose in the depot GnRH agonist protocol for the early follicular phase

**DOI:** 10.3389/fendo.2025.1542736

**Published:** 2025-03-27

**Authors:** Wenqian Fan, Tian Ye, Linqing Du, Lifeng Tian, Huijuan Kong

**Affiliations:** ^1^ Reproductive Medical Center, Henan Province Key Laboratory for Reproduction and Genetics, The First Affiliated Hospital of Zhengzhou University, Zhengzhou, China; ^2^ Reproductive Medicine Center, Jiangxi Maternal and Child Health Hospital, Nangchang, China

**Keywords:** the early follicular phase depot GnRH agonist (EFDGa) protocol, ovarian responsiveness, ideal FSH starting dose, nonogram model, internal and external validation

## Abstract

**Background:**

Each controlled ovarian hyperstimulation(COH) protocol has its own unique mechanism and hormone pattern. The depot GnRHa protocol has a deeper down-regulation effect and favorable clinical pregnancy rates. The predictive model of the optimal follicle-stimulating hormone (FSH) starting dose in the early follicular phase depot GnRH agonist (EFDGa) protocol has not been reported. Our study was made to explore predictive indicators for determining the optimal FSH starting dose in patients undergoing ovarian stimulation with the EFDGa protocol in assisted reproductive technology (ART), and to develop and validate a nomogram prediction model for the starting dose of FSH.

**Methods:**

This retrospective study included 2733 cycles who underwent fresh cycle transplantation at two large teaching hospitals in China from January to December 2022: center 1 (Reproductive Medicine Center of first affiliated Hospital of Zhengzhou University) provided the data for modelling (n = 938) and internal testing (n = 400), and center 2 (Reproductive Medicine Center of Jiangxi Maternal and Child Health Hospital) provided the data for external testing (n = 1109). Patient demographics, including age, anti-Mullerian hormone (AMH) levels, baseline endocrine profile, and body mass index (BMI), along with information on ovulation stimulation, were collected. Univariate and multivariate linear regression models were used to identify factors influencing the FSH starting dose. A nomogram for the ideal FSH starting dose was developed based on these factors and validated internally and externally. Bland and Altman plots and paired t-tests were conducted to verify the concordance between groups.

**Results:**

Multivariate analysis revealed that patient age, BMI, basal FSH, AMH, and antral follicle count (AFC) were indicators of FSH starting dose. The regression model for predicting FSH starting dose was determined as: Initial FSH dose = 62.957 + 1.780*AGE(years) +4.927*BMI (kg/m²) +1.417*bFSH (IU/ml) - 1.996*AFC - 48.174*AMH (ng/ml). Bland and Altman analysis showed good agreement in the internal validation (bias: 0.583, SD of bias: 33.07IU, 95%LOA: -69.7 to 68.5IU b). Furthermore, validating the model on external cohort (center 2) confirmed that nomogram prediction model is an accurate predictor of FSH starting dose ((bias: -1.437, SD of bias: 38.28IU; 95%LOA: -80.0 to 77.1IU).

**Conclusions:**

We established a model for effectively predicting the ideal FSH starting dose, with the nomogram model providing an intuitive representation of the data. The predictive model demonstrates practical utility, effectively initiating a proper ovarian response and preventing adverse ovarian reactions or the occurrence of ovarian hyperstimulation syndrome. As more IVF cycles are being generated in the future, this model will be valuable in clinicians using basic parameters to assess proper initial dose of FSH.

## Introduction

Recently, the EFDGa protocol has gained widespread use in ART. This protocol is favored for its ability to enhance oocyte maturity and utilization (1, 2). It achieves this by increasing the number of endometrial pinopodes and the expression of related factors, thereby improving endometrial receptivity, reducing concentrations of pelvic inflammatory mediators, and enhancing the pelvic environment, ultimately promoting favorable conditions for embryo implantation (3). This approach has been associated with increased live birth rates in each fresh embryo transfer (ET) cycle (4, 5), with no significant changes in ovarian hyperstimulation syndrome (OHSS) incidence (6, 7). Clearly, EFDGa protocol is a friendly stimulation protocol for infertility patients.

To determine The FSH initiation dose is a critical step in managing controlled ovarian hyperstimulation (COH). Inadequate initiation doses may artificially induce unexpected poor response of Poseidon type, leading to poor follicular growth, significantly reduced oocyte retrieval, and increased cycle cancellation rates (8). Conversely, excessively high initiation doses may elevate the risk of OHSS, and elevated estradiol levels may impact endometrial receptivity (9). Additionally, excess gonadotropins usage may increase progesterone levels during ovulation induction, leading to higher cancellation rates for fresh cycles or decreased pregnancy rates due to asynchronous endometrial development; complications such as bleeding during egg retrieval and the high levels of ovarian stimulation hormones are associated with oocyte chromosomal abnormalities (10). Therefore, an appropriate FSH initiation dose is crucial in improving clinical outcomes in patients undergoing assisted reproductive fertility treatments.

In recent years, more and more experts are willing to apply the concept of “Individualization” rather than “One size fit all” in IVF in ovarian stimulation (11–13). Domestic and international guidelines recommend varying initial doses based on age and accompanying conditions (14, 15). With the development of artificial intelligence (AI), there were several starting-dose predicted models focusing on GnRH antagonist protocols (14, 16), progestin primed ovarian stimulation protocol protocols (17), classical long agonist protocols (18, 19). Lately, comparison will be made in the discrepancy between the nomogram and clinical practice. A non-inferiority study protocol for a multi-center randomized controlled trial was conducted to test Personalizing the first dose of FSH for IVF/ICSI patients through machine learning *vs* the clinician following standard practice (20). Finally, the goal is to get a cost-effective starting dose that will obtain an optimal number of oocytes.

Previous researchers have shown interest in prediction models for determining the starting dose, with numerous models developed for GnRH antagonist and long agonist protocols. Each COH protocol has its own unique mechanism and hormone pattern. The depot GnRHa has a deeper down-regulation effect and a different hormone pattern, compared to the antagonist protocol and short-acting down-regulation protocols (1). The hypothesis underlying this protocol was that using an appropriately mild starting FSH dose might achieve optimal laboratory outcomes while reducing the amount of starting and total FSH given to the patient, which could help to reduce unnecessary ovarian hyperstimulation, and lower the costs associated with IVF, especially in populations with high ovarian reserves and low body weight. Those models above mentioned were inappropriate to use in the EFDGa protocol. Constructing a predictive model for the optimal FSH initial dose in the EFDGa protocol is of significant clinical importance. It could improve the effectiveness of ovulation induction, reduce complication rates, shorten the time to achieving a live birth, save on treatment costs for patients, and enhance overall prognosis. In this study, we aimed to utilize the multivariate linear regression method to construct a model predicting the optimal FSH initial dose, aiming to increase the first-time success rate, effectively shorten the adjustment period, alleviate patients’ financial burdens, and improve overall prognosis. The model was trained using basal characteristic parameters collected from a specific center, and its performance was evaluated on datasets from internal and external centers.

## Materials and methods

### Study population

**Figure 1:**
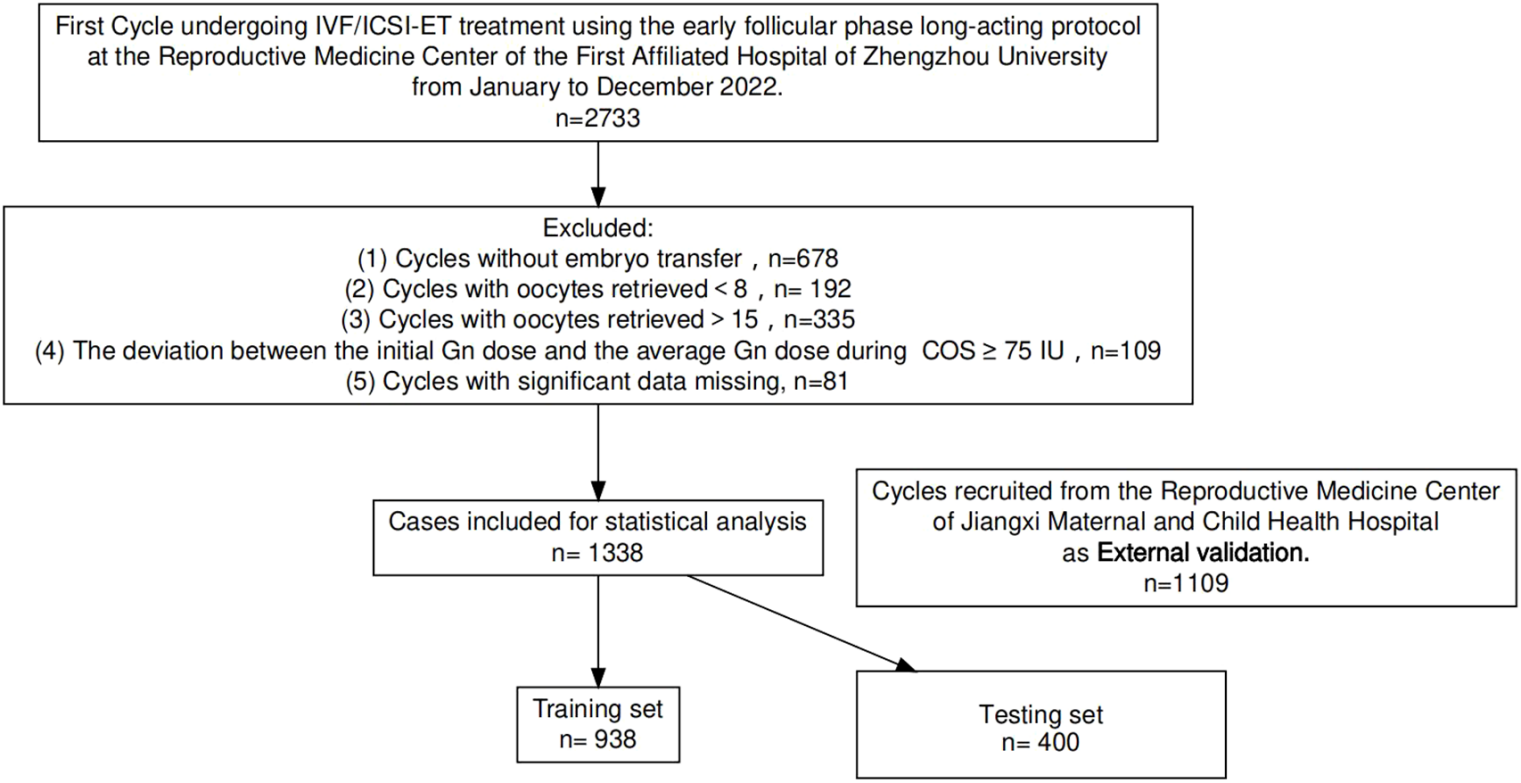
Flowchart of patient screening.

A retrospective analysis was conducted on clinical data from 1338 cycles undergoing *in vitro* fertilization-embryo transfer (IVF-ET) or intracytoplasmic sperm injection-embryo transfer (ICSI-ET) treatments using the early follicular phase long-acting protocol at the Reproductive Medicine Center of the First Affiliated Hospital of Zhengzhou University from January to December 2022. 1109 cycles were recruited in the Reproductive Medicine Center of Jiangxi Maternal and Child Health Hospital. patients were selected if the following inclusion criteria were satisfied: (1) Use of the EFDGa protocol (2)The dispense between the practice initial starting dose and the average dose throughout the cycle were limited to less than 75IU; (3) Oocytes retrieved between 8 and 15; (4) This was the patient’s first oocyte retrieving cycle (5); Cycle outcome was embryo transferred. Exclusion criteria: (1) Cycles with significant data missing; (2) Patients with chromosomal abnormalities, as shown in **Figure 1**. Written informed consent was collected from all participants prior to the ART procedures. All methods were performed in accordance with the Declaration of Helsinki (1983). The study was approved by the Institutional Ethical Committee of first affiliated Hospital of Zhengzhou University.

### Clinical data

The clinical characteristics of the patients were collected, including age, BMI, and AFC; laboratory biochemical examination indicators before ovulation induction, such as AMH and basic endocrine conditions; indicators during controlled ovarian stimulation (COS), including the FSH initial dose, duration of GN use, and the total GN dose; and ovulation induction outcome indicators, such as the total number of oocytes retrieved.

### Controlled ovarian stimulation protocol

The routine EFDGa protocol was employed, with administration of the long-acting GnRH agonist (Diphereline, Ipsen, France) 3.75 mg on menstrual cycle days 2−4 for down-regulation. To achieve the pituitary down-regulation criteria (FSH < 5 U/L, luteinizing hormone [LH] < 5 U/L, estradiol [E2] < 50 pg/mL, endometrial thickness < 5 mm, and no functional ovarian cysts), FSH stimulation (75–300 IU/day) was initiated. During the medication process, FSH dose was adjusted according to ovarian response and hormone levels. Triggering was performed when at least one dominant follicle reached a diameter of ≥20 mm or three follicles reached a diameter of ≥18 mm, using recombinant human chorionic gonadotropin (hCG; 250μg, Ovitrelle, Merck Serono S.p.A., UK) and/or hCG 2000IU (Zhuhai Livzon Pharmaceutical Group) injection. Ovum retrieval was conducted 37h post-triggering under transvaginal ultrasound guidance, followed by routine luteal phase support. Conventional IVF or ICSI was performed based on patient conditions.

### Statistical analysis

A predictive nomogram for gonadotropin starting dose was developed based on the results of multivariate analysis and the formula for numbers of oocytes. Included patients were treated as “standardized cycles” by specialist clinician in reproductive medicine. They have retrieved appropriate number of oocytes. On the other hand, the dispense between their actual initial starting dose and the average dose throughout the cycle were limited to less than 75IU. The actual starting dose in standardized cycles were considered as the optimal starting dose to build the model and test the performance of the model.

Complete case analysis was employed, retaining only those without any missing variables for analysis. Data analysis and a nomogram model was conducted using R-4.3.2. Quantitative data were presented as mean ± standard deviation, and group comparisons were performed using t-tests. Count data were expressed as composition ratios or percentages, and group comparisons were conducted using chi-square tests. With the use of the training set, relevant factors influencing the FSH initiation dose were identified through univariate analysis and multiple linear regression models (based on clinical meaning and the significance [P < 0.05] of variables in univariate analysis). Pearson’s correlation was used to assess the correlation of FSH initial dose between the model prediction results and the clinically ground truths. Additionally, agreement between the two dose was explored using Bland–Altman plots with 95% limits of agreement on datasets from internal and external centers. A significance level of p< 0.05 indicated statistical significance. All statistical graphics was performed using Prism 8.0.2 (GraphPad Software, USA).

## Results

### Demographics and general characteristics

**Table 1** displays essential information collected from the two medical centers. The clinical baseline characteristics included in the study were female age, duration of infertility, body mass index (BMI), baseline hormone levels (bFSH, bE2, bLH, anti-Mullerian hormone (AMH)), antral follicle count (AFC), FSH initiation dose, and total gonadotropin dose. A comparison of clinical baseline data between the training and testing sets in two centers is also presented in **Table 1**. The results indicate that, apart from the BMI and AFC values being significantly different in the external validation set compared to the training set (P < 0.001 and P = 0.002, respectively), there were no other statistically significant differences in baseline data between the three groups (P ≥ 0.05).

**Table 1:** Patient demographics and baseline characteristics from two centers.

Characteristic	Internal validation		p-value^2^	External validation		
	Training Set, (N = 938, from Centre 1)	Testing Set, (N = 400, from center 1)		Training Set, (N = 938, from Centre 1)	Validation Set, (N = 1109, from center 2)	p-value^2^
AGE (years)	30.9 ± 4.0	30.7 ± 4.3	0.367	30.9 ± 4.0	30.8 ± 4.1	0.755
BMI (kg/m2)	23.2 ± 3.3	23.1 ± 3.1	0.698	23.2 ± 3.3	22.0 ± 3.1	<0.001
AFC (n)	15.0 ± 5.7	14.7 ± 5.6	0.077	15.0 ± 5.7	14.6 ± 5.2	0.002
AMH (ng/ml)	3.61 ± 2.36	3.33 ± 2.09	0.036	3.61 ± 2.36	3.78 ± 3.79	0.204
bFSH (IU/L)	6.44 ± 1.81	6.61 ± 1.92	0.131	6.44 ± 1.81	6.47 ± 2.53	0.772
bLH (IU/L)	5.9 ± 5.5	5.5 ± 3.4	0.145			
bE2 (pg/ml)	44 ± 38	46 ± 45	0.091			
bP (ng/ml)	0.36 ± 0.65	0.47 ± 1.32	0.138			
Initial FSH dose (IU)	158 ± 51	163 ± 51	0.176	158 ± 51	156 ± 55	0.390
days of GN (d)	12.97 ± 1.72	12.89 ± 1.82	0.486			
Total gonadotropin dose(IU)	2,566 ± 830	2,614 ± 873	0.355			
Oocyte (n)	11.51 ± 2.19	11.41 ± 2.25	0.470	11.51 ± 2.19	11.38 ± 2.18	0.181

Values are presented as mean ± standard deviation.

Center 1, Reproductive Medicine Center of the First Affiliated Hospital of Zhengzhou University; Center 2, Reproductive Medicine Center of Jiangxi Maternal and Child Health Hospital.

BMI, body mass index; AFC antral follicle count, AMH, anti-Müllerian hormone; bFSH, basal follicle-stimulating hormone, bLH, basal luteinizing hormone; bE2, basal estradiol; bP, progesterone.

### Univariate and multivariate correlation analysis of factors influencing appropriate FSH initiation dose

**Figure 2:**
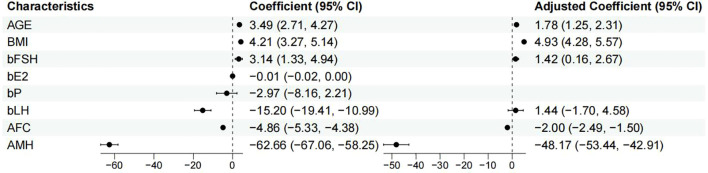
Forest plot shows univariate and multivariate correlation analysis of factors influencing appropriate FSH initiation dose.

The predictor variables AMH and bLH displayed a right-skewed distribution, which approached a normal distribution after log transformation. Univariate and multivariate correlation analysis was performed with the training cohort, as represented in **Table 2**. The multivariate regression analysis revealed significant associations between several independent variables and FSH initiation dose. Age was positively associated with FSH initiation dose (β = 1.780; 95% CI, 1.246 to 2.314; p < 0.001), indicating that each additional year of age corresponded to an increase in FSH initiation dose. Similarly, higher BMI was strongly associated with greater FSH initiation dose (β = 4.927; 95% CI, 4.282 to 5.571; p < 0.001). Basal FSH levels also showed a positive relationship with FSH initiation dose (β = 1.417; 95% CI, 0.160 to 2.673; p = 0.027), while basal LH levels did not reach statistical significance (β = 1.437; 95% CI, -1.701 to 4.576; p = 0.370). In contrast, both AFC and AMH were inversely associated with FSH initiation dose. Each unit increase in AFC was linked to a decrease in FSH initiation dose (β = -1.996; 95% CI, -2.494 to -1.497; p < 0.001), and higher AMH levels were associated with a substantial reduction in FSH initiation dose (β = -48.174; 95% CI, -53.436 to -42.913; p < 0.001), as represented in **Figure 2**.

**Table 2:** Univariate and multivariate analysis of influencing factors in training set.

Characteristic	Univariable				Multivariable		
	N	Beta	95% CI^1^	p-value	Beta	95% CI^1^	p-value
AGE	938	3.488	2.710, 4.265	<0.001	1.780	1.246, 2.314	<0.001
BMI	938	4.205	3.271, 5.140	<0.001	4.927	4.282, 5.571	<0.001
bFSH	938	3.137	1.332, 4.941	<0.001	1.417	0.160, 2.673	0.027
bE2	938	-0.007	-0.015, 0.001	0.123			
bP	938	-2.974	-8.158, 2.211	0.261			
bLH	938	-15.200	-19.414, -10.986	<0.001	1.437	-1.701, 4.576	0.370
AFC	938	-4.857	-5.333, -4.379	<0.001	-1.996	-2.494, -1.497	<0.001
AMH	938	-62.656	-67.062, -58.251	<0.001	-48.174	-53.436, -42.913	<0.001

^1^CI = Confidence Interval.

### Model development

**Figure 3:**
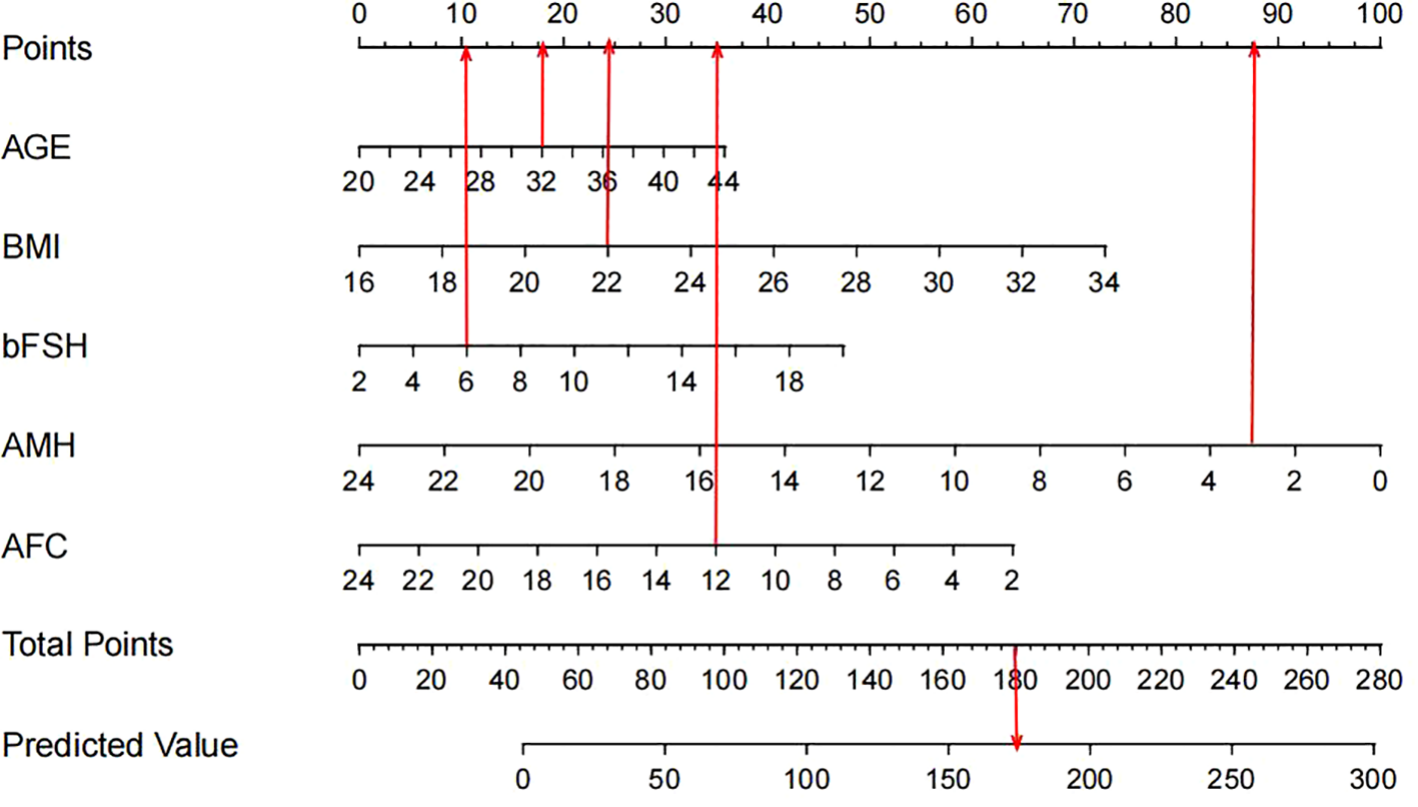
Nomogram for prediction of FSH initial dosage.

This study’s predictive model incorporates five independent influencing factors, and an equation was established as: Initial FSH dose = 62.957 + 1.780*AGE(years) +4.927*BMI (kg/m²) +1.417*bFSH (IU/ml) −1.996*AFC −48.174*AMH (ng/ml) This regression equation revealed that the FSH initiation dose was positively correlated with age, BMI, and bFSH, AMH and AFC are negative factors influencing the FSH initiation dose which decreasing as AMH and AFC increase. Based on the nomogram, as shown in **Figure 3**, we obtained the corresponding score for each predictive factor and located the actual values for each variable on the bar chart. For instance, an age of 32 years might correspond to a score of 18 on the graph, while a BMI of 22 kg/m² could also correspond to a score of 25, a bFSH of 6IU/ml correspond to 10, a AMH of 3 ng/ml correspond to 90, and a AFC of 12 correspond to 35. Repeat these steps for all variables in the graph, sum up the scores to get the total points of 178, locate the corresponding position in the “Total Points” column. Move vertically from the “Total Points” row to the “Predicted Value” scale, finding the corresponding predicted value of 175IU. The predictive model demonstrated a R^2^ value of 0.608, which means the developed model above explained approximately 60% of the variability in the FSH prediction, and a Root Mean Squared Error (RMSE) of 12.190, as demonstrated in **Table 3**.

**Table 3:** Assessment of model performance in the validation group compared to the Modelization group.

Parameter	Modelization group (n =938)	Internal Validation group (n =400)	External Validation group (n =1109)
R^2^	0.608	0.543	0.443
MAE [95% CI]	6.242 [2.365, 10.119]	8.036 [2.520, 13.552]	15.706 [5.962, 25.450]
RMSE	12.190	14.665	31.754
Paired t-test		0.790	0.440
Bland–Altman plot			
bias (SD)		-0.583(33.07)IU	-1.437 (38.28)IU
95%LoA		-69.7 – 68.5 IU	-80.0 – 77.12 IU

R2, R-squared; MAE, Mean Absolute Error; RMSE, Root Mean Squared Error; Bland-Altman plot, a tool used to evaluate the consistency between two independent measurement methods. 95%LoA, 95%limits of agreement.

### Internal and external validation of the model

**Figure 4:**
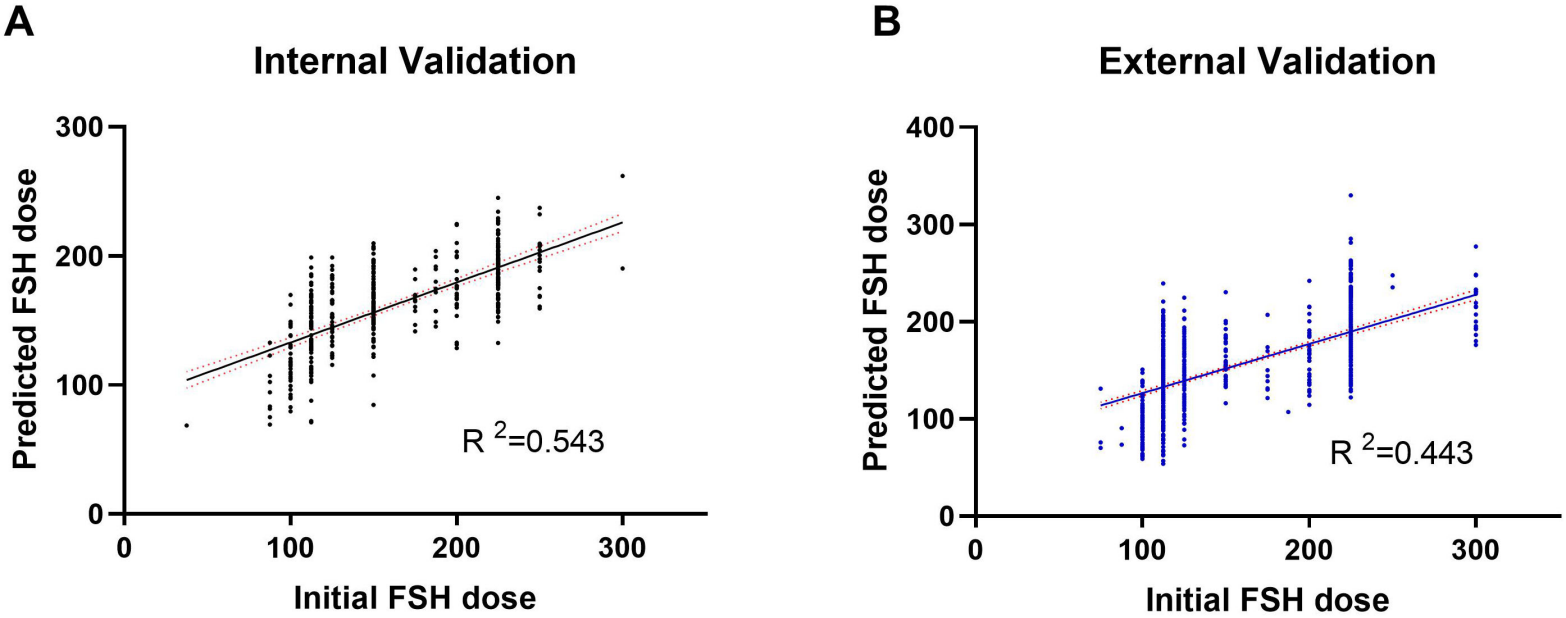
Actual *vs*. Predicted plot of FSH initial dosage in both the internal **(A)** and external **(B)** validation.

**Figure 5:**
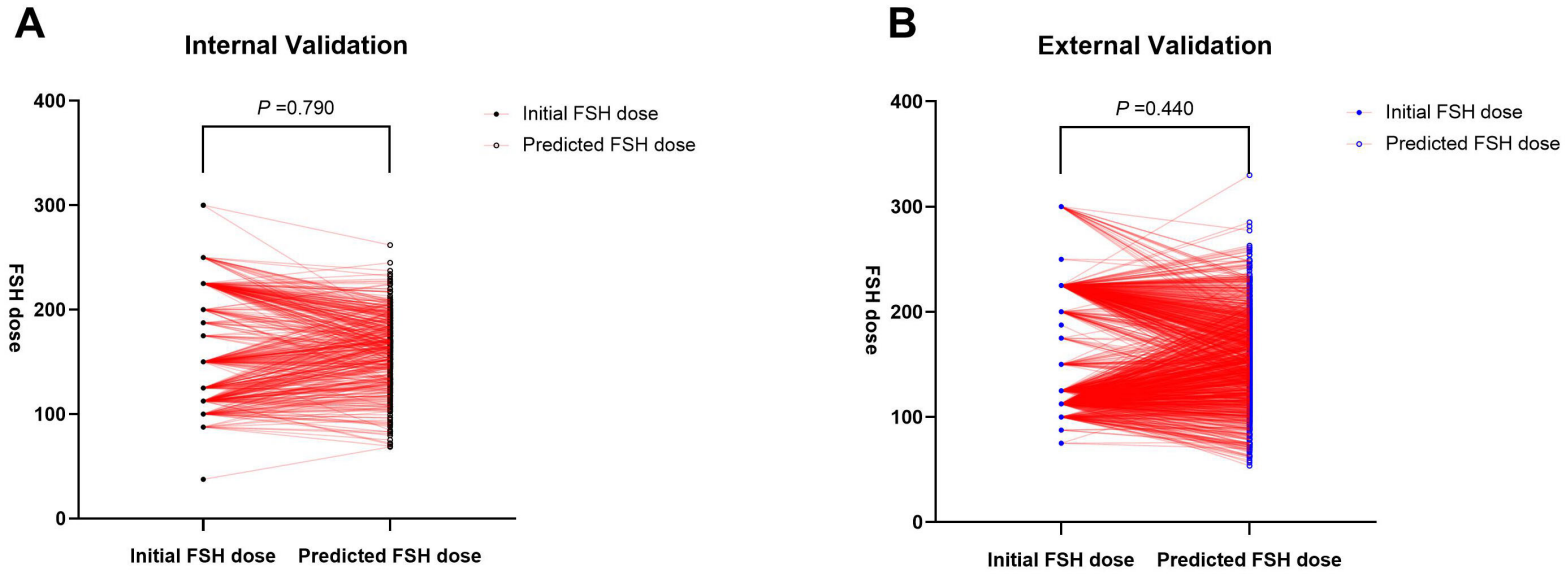
Paired t-test for predicted and actual FSH initial dosage in both the internal **(A)** and external **(B)** validation.

**Figure 6:**
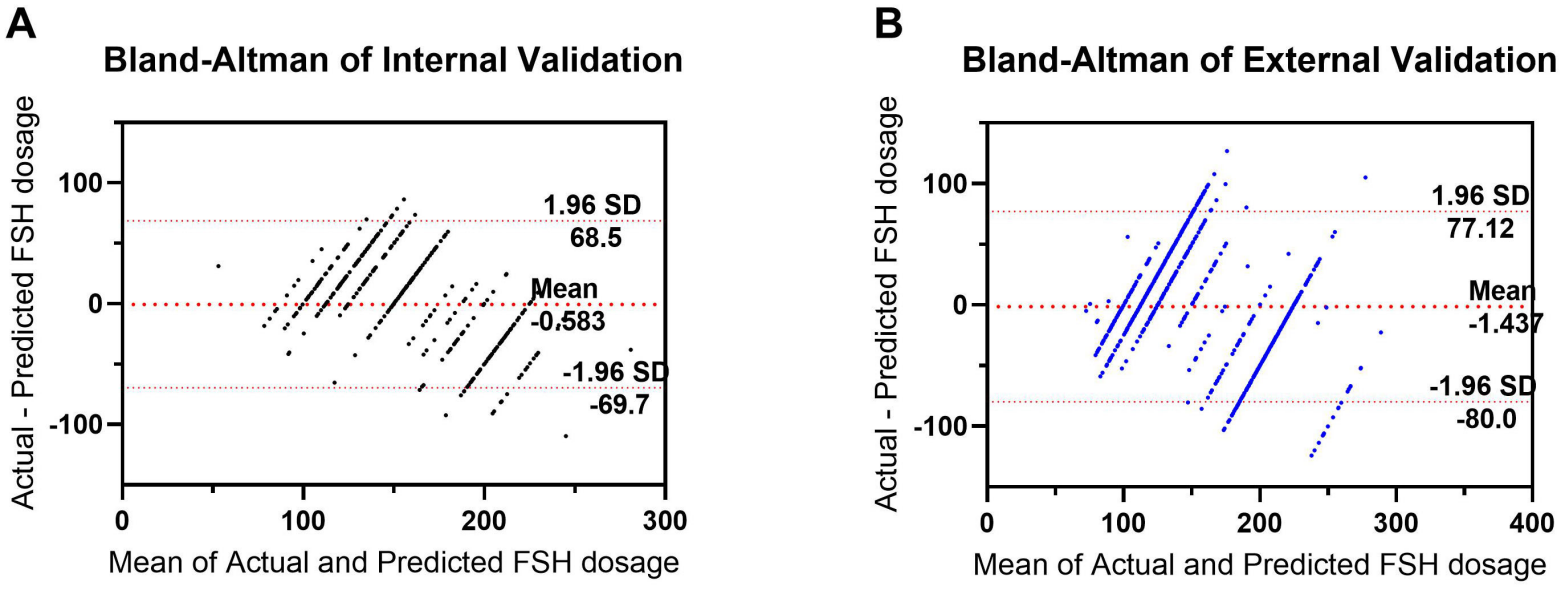
Bland–Altman plot for predicted and actual FSH initial dosage in both the internal **(A)** and external **(B)** validation. The differences between the Actual and Predicted groups are plotted against the averages of the two groups.

A random selection of 400 patients from center 1 was chosen for internal validation. The fit curve (**Figure 4A**) between the predicted FSH dosage and the ground-truth FSH initiation dosage resulted in a R^2^ value of 0.543, a MAE value of 6.242[2.520 to 13.552], and a RMSE value of 12.190. The paired t-test for predicted and actual dosage yielded a p-value of 0.790 (**Figure 5A**), while the Bland–Altman plot revealed a bias of -0.583 (SD, 33.07IU, 95% limits of agreement, -69.7 to 68.5IU ) (**Figure 6A**). For external validation, the fit curve (**Figure 4B**) for predicted and ground-truth FSH dosage resulted in a R^2^ value of 0.443, a MAE value of 15.706[5.962, 25.450], and a RMSE value of 31.754. The paired t-test for predicted and actual dosage yielded a p-value of 0.440 (**Figure 5B**), while the Bland–Altman plot revealed a bias of −1.437 (SD, 38.28IU; 95% limits of agreement, -80.0 to 77.1IU ) (**Figure 6B**). These results suggested the good value of reliability and repeatability of this concluded model, and demonstrated the improved clinical benefits provided by the predictive model.

## Discussion

In this study, we investigated the predictive indicators for determining the optimal FSH starting dose in patients undergoing superovulation treatment with the EFDGa protocol and constructed a nomogram prediction model. This method allowed the automatic assessment of FSH starting dose in patients using female age, BMI, baseline FSH, AMH, and AFC which were basal clinical parameters as inputs. To evaluate the method, the nomogram was trained using the data from center 1, and independent testing was conducted using test sets from both internal and external centers separately. Model reliability was confirmed in the training and both internal and external validation sets using Bland and Altman plots and paired t- test.

However, the performance of the model in external validation (R² = 0.443) is slightly lower than that in internal validation (R² = 0.543), and we believe that this discrepancy may be caused by the following factors:1. Differences in data characteristics: For instance, BMI and AFC values were significantly different in the training set compared to the external validation set (P < 0.001 and P = 0.002, respectively), which may affect the prediction performance of the model; 2. The absence of key predictor variables: For instance, genetic markers such as FSH receptor polymorphisms and physiological factors like the ovarian blood flow index could influence the prediction of FSH dose.

In ART cycles, the primary objective of ovarian stimulation is to induce the development of multiple follicles, thereby obtaining an optimal quantity of viable oocytes to enhance the pregnancy rate. According to the follicular threshold concept proposed by Brown (21), elevating FSH concentration above the threshold by 10% to 30% is sufficient to stimulate normal follicular development. Exogenous supra-physiological levels of FSH play a crucial role in inducing the development of multiple follicles, allowing the recruited follicles to continue maturing. However, there is significant variability in the FSH sensitivity threshold among individuals. Our study revealed that the appropriate FSH initiation dose is not only correlated with age and parameters reflecting ovarian reserve such as AMH, bFSH, and AFC but also associated with BMI. The use of FSH initial doses in the long-acting protocol for the follicular phase currently lacks consensus and guidelines among experts.

Due to the advantages of the EFDGa protocol, characterized by high endometrial receptivity in fresh cycles, a gentle step-up approach is commonly employed. Although this may reduce the average number of retrieved oocytes, there is a significant improvement in fertilization rates (2PN), embryo implantation, clinical pregnancy, and high-quality embryo formation (22). However, for patients whom initial FSH dose does not reach the threshold or who exhibit a suboptimal ovarian response, timely adjustment of FSH dosage during the stimulation process is equally crucial. Hence, we selected an inclusion criterion of FSH increment dosage less than 75IU. Several studies have confirmed that obtaining 15 oocytes maximizes live birth rates in fresh embryo transfer cycles (23). Steward et al., analyzing 256,381 cycles, identified that retrieving more than 15 oocytes was the optimal predictor for the risk of OHSS (24). In studies of long-acting protocols, various centers have suggested that the range of retrieved oocytes between 10−17 and 6−20 achieves satisfactory clinical pregnancy rates and lower rates of severe OHSS in fresh embryo transfer cycles (22, 25), thereby reducing the time to achieving a live birth. Therefore, in this study, we defined the optimal oocyte retrieval range as 8−15.

Several formulas or models predicting FSH initiation doses for different ovarian stimulation protocols have been reported globally. Antonio La Marca et al. (26) identified three relevant factors (age, AFC, FSH) and developed a nomogram for predicting FSH initiation doses. Unfortunately, their study focused on the luteal phase protocol, employing a step-down dosage pattern. Additionally, due to ethnic differences, this model may not apply to the Chinese population. In China, two studies have investigated FSH initiation doses for patients using antagonist protocols (16, 27), but they primarily targeted patients with polycystic ovary syndrome (PCOS) rather than those with normal or low ovarian reserve. PCOS, characterized by high ovarian reserve and response, makes their model unsuitable for patients with normal or low ovarian reserve. Our multifactorial regression model revealed that patient age, BMI, basal FSH, AMH, and AFC are essential reference factors for FSH initiation doses, aligning with clinical knowledge. For patients with high ovarian reserve, this model can more accurately predict ovarian response, aiding in the development of personalized ovulation induction protocols and reducing the risk of OHSS. Additionally, some earlier studies that used AMH or AFC alone predicting ovarian reserve (28) or nomograms that did not include BMI as a variable (18, 29) are consistent with our findings.

Age has long been under scholarly discussion as a predictor for the initiation dose of gonadotropin (FSH) in ART. The decline in fertility with age is primarily characterized by a reduction in the number of follicles, a diminished response of the ovaries to exogenous gonadotropin, and a significant decrease in pregnancy and live birth rates. To address these physiological changes, ovarian stimulation typically employs high-intensity FSH stimulation for elder individuals, while younger individuals with high ovarian reserves may require more precise low-dose FSH stimulation. The positive correlation trend with age in this model aligns with previous research (30, 31). Interestingly, some predictive models do not incorporate age as a factor, potentially due to their target population being PCOS patients who generally possess higher ovarian reserves. In such cases, bLH may have a more pronounced impact on FSH initiation doses than age (27). Alternatively, models using the progestin primed ovarian stimulation (PPOS) protocol may diminish the influence of age by initiating FSH more intensively (17).

This study confirms a close correlation between FSH initiation doses and ovarian reserve. The decrease in ovarian reserve was manifested by a reduction in AFC and an elevation in FSH levels. This finding aligns with expressions in other models (17, 26). Recently, AMH has been considered more accurate than basal FSH in predicting ovarian reserve and is widely recognized as one of the simplest, most sensitive, and reliable indicators for evaluating ovarian reserve (32). There is generally a positive correlation between AMH and AFC, although some studies show a 30% inconsistency between the two indicators (33, 34). In clinical practice, predicting ovarian responsiveness with a single parameter is challenging. Therefore, clinicians often conduct multiple assessments and utilize predictive models combining various parameters to enhance the effectiveness of predicting ovarian responsiveness. In contrast to the study by Simanfei (27), we did not include LH as an influencing factor after multifactorial analysis in this study. The rationale behind this decision lies in the diverse characteristics of the included populations, with some PCOS patients exhibiting significantly higher LH levels than FSH. However, in the general population, no such inclination in baseline LH levels was observed. This aligns with the consistent exclusion of LH as a modeling factor in most studies involving the general population.

We observed a significant correlation between BMI and the initiation dose of gonadotropin (FSH). Body weight is one of the factors that can interfere with the secretion of gonadotropins, and obesity is associated with ovarian dysfunction (35). The serum levels of gonadotropins entering the body are directly influenced by body weight. Therefore, body weight is also considered an indicator for predicting the initial dose. Some studies indicate that, compared to weight and body surface area, BMI is a more important factor for predicting the number of retrieved oocytes (27). BMI specifically influences the adjustment of ovulation-inducing drugs in patients with PCOS (36) and pregnancy and live birth outcomes (37).

Recently, nomograms have been applied in various fields of reproductive medicine to predict the likelihood of human embryo euploidy (38, 39), oocyte retrieval (40), fertilization failure (41), as well as predicting live birth rates for different types of patients seeking fertility assistance (42, 43). Drawing a nomogram to predict ovarian response and the optimal FSH initiation dose is a simple, efficient, and feasible approach. To our knowledge, this study is the first to develop a nomogram for predicting the FSH initiation dose in the EFDGa protocol.

The strength of this model lies in the precise construction of a nomogram for the initiation dose of FSH based on clinical and biological variables. This nomogram is designed for individualized prediction of the initial FSH dose in patients outputting with normal responses undergoing IVF/ICSI. The model is weighted, considering the different weights of various predictive factors, enabling a more objective and personalized prediction of the FSH initiation dose. Furthermore, this study had an ample sample size, and both internal and external validations were conducted, indicating the model’s stable predictive value. The factors included in this model are straightforward and easily measurable in clinical practice.

In this study, we compared the FSH dose predicted by the model with the dose actually prescribed by experienced physicians to evaluate the model’s utility in practical clinical decision-making. The results indicated no significant difference (the p values of the paired t-test were 0.790 and 0.440, respectively, in internal and external validation). This suggests that the model’s predictions are close to actual clinical decisions and can provide a valuable reference for clinical practice. This data-driven prediction tool can assist experienced doctors in optimizing treatment plans and also aid novice doctors in rapidly accumulating experience and improving their clinical decision-making skills. The results of this study are poised for straightforward clinical implementation and hold promise for wider clinical adoption.

However, this study has several limitations. First, ovarian responsiveness is largely influenced by individual factors, and the polymorphism of FSH and its receptor genes is crucial for the individualized use of FSH doses in treatment plans. Patients with similar baseline characteristics may exhibit significant differences in response to gonadotropin drugs. Due to the limited clinical adoption of genetic receptor polymorphism testing, this aspect was not thoroughly analyzed in this study. Second, the advantage of the long protocol for the early follicular phase lies in achieving a higher pregnancy rate with fresh cycle embryo transfer. However, this study included patients with 8−15 retrieved eggs as modeling criteria without considering the maturity of eggs, embryo quality, and pregnancy outcomes. Therefore, the predictive value of this model for pregnancy outcomes is limited. Third, the FSH used in this study included recombinant human follicle-stimulating hormone alpha (Merck Serono SA Aubonne Branch, Switzerland), recombinant human follicle-stimulating hormone beta (Merck Sharp & Dohme, USA), and urinary follicle-stimulating hormone (Livzon Pharmaceutical Group, China). These drugs may have varying biological potencies, which could impact the model results. And different laboratories may use different methods to measure AMH levels, and various assay methods have different specificities and sensitivities, potentially affecting the generalizability of this model. Finally, our model’s accuracy appears to have experienced a mild decline during external validation, likely due to population heterogeneity. Its applicability in other regions or among different ethnic populations requires further validation.

Therefore, to enhance the model’s universality and predictive power, we intend to incorporate additional predictive variables, such as genetic markers and ovarian blood flow indices, along with other potential predictors to enrich its predictive capabilities. We also plan to expand the scope of validation by verifying the model against more diverse external datasets, including those from various regions and populations, to further assess its universality. Additionally, we aim to optimize the model’s structure by experimenting with more complex statistical methods, such as machine learning models, to capture more intricate nonlinear relationships.

## Conclusions

This study investigated the ability of an a clinically practical nomogram to assess the appropriate starting dose of gonadotropin in patients with normal responses using the long-acting downregulation protocol for the early follicular phase for the first cycle. Our work extends the automated detection of FSH dose, and our method exhibits moderate performance and demonstrates great potential for clinical development. To improve upon our work, future research should expand the dataset to include more kind of special patients and further widespread application in clinical practice to guide clinical decisions.

## Data Availability

The raw data supporting the conclusions of this article will be made available by the authors, without undue reservation.
